# Bio-efficacy and physical integrity of piperonylbutoxide coated combination net (PermaNet^®^ 3.0) against pyrethroid resistant population of *Anopheles gambiae* s.l. and *Culex quinquefasciatus* mosquitoes in Ethiopia

**DOI:** 10.1186/s12936-019-2641-1

**Published:** 2019-07-04

**Authors:** Abaynesh Birhanu, Abebe Asale, Delenasaw Yewhalaw

**Affiliations:** 1 Department of Biology, College of Natural Sciences, Jimma University, Jimma, Ethiopia; 2 Faculty of Health Sciences, School of Medical Laboratory Sciences, Jimma University, Jimma, Ethiopia; 3 Tropical and Infectious Diseases Research Center, Jimma University, Jimma, Ethiopia; 4 International Center of Insect Physiology and Ecology, Addis Ababa, Ethiopia

**Keywords:** Bio-efficacy, PermaNet^®^ 3.0, Net integrity, Cone bioassay, Mosquito, Proportionate hole index, Ethiopia

## Abstract

**Background:**

PermaNet^®^ 3.0 is a deltamethrin-treated combination long-lasting insecticidal net with the addition of synergist piperonylbutoxide (PBO) on its roof section. It is designed to overcome the challenge posed by pyrethroid resistant vector populations against mainstream long-lasting insecticidal nets impregnated with pyrethroids only. The objective of this study was to determine insecticide resistance status of Anopheline and Culicine mosquitoes, to evaluate the bio-efficacy of PermaNet^®^ 3.0 nets and to assess household factors affecting the physical integrity of PermaNet^®^ 3.0 after 3 years of use.

**Methods:**

Insecticide susceptibility test was conducted using the WHO tube test. Bio-activity of PermaNet^®^ 3.0 samples was evaluated using the WHO cone bioassay. Cross-sectional survey was conducted on 150 randomly selected households from two districts to determine household factors affecting net utilization. One hundred fifty PermaNet^®^ 3.0 nets were randomly collected from the community with replacement after 3 years of deployment and physical integrity of each net was assessed.

**Results:**

Both *Anopheles gambiae* sensu lato and *Culex quinquefasciatus* developed resistance against permethrin and deltamethrin. However, following pre-exposure to synergist PBO the susceptibility of mosquito population increased to both permethrin (from 39% without to 92% with PBO against *An. gambiae* and from 28% without to 94% with PBO against *Culex quinquefasciatus*) and deltamethrin (from 52% without to 99% with PBO against *An. gambiae* and from 43% without to 98% with PBO against *Culex quinquefasciatus*). Eighty percent (80%) mortality was recorded in wild population of *An. gambiae* s.l. exposed to unused PermaNet^®^ 3.0, but its bioactivity subsequently declined as washing frequency increased from 0 to 20. The PBO coated roof section of unused PermaNet^®^ 3.0 resulted in higher mosquito mortality (100%) compared to the side panels without PBO (85%). House structure, cooking and washing habits, and damage due to household pests were cited as determinants associated with bed net deterioration. Bed net proportionate hole index (pHI) was ranged from 0 to 6064. Of the 150 PermaNet^®^ 3.0 nets assessed 80, 29 and 41 were considered as ‘good’, ‘acceptable’ and ‘too torn’, respectively.

**Conclusions:**

The bio-efficacy evaluation of PermaNet^®^ 3.0 from Jimma area, southwestern Ethiopia showed moderate efficacy against pyrethroid resistant population of *An. gambiae* and *Culex quinquefasciatus.* Thus, NMCPs in parallel to deployment of LLINs, should implement timely insecticide resistance management and integrated vector management strategies to slowdown the evolution and further spread of insecticide resistance. Household factors such as, housing conditions, open flame fire used for cooking and rodent attack were identified as factors contributing to the observed reduced bed net physical integrity in the study area. Universal coverage of bed nets should be accompanied with community awareness creation and training on net utilization and handling.

## Background

Malaria control efforts remain a priority agenda globally and particularly in Africa where the disease claims hundreds of thousands of lives each year [1]. Long-lasting insecticidal nets (LLINs), indoor residual spraying (IRS) and environmental management are the most widely used tools for malaria vector control worldwide [1–3]. In Ethiopia, LLINs, IRS, environmental management and rapid diagnostic tests (RDTs) coupled with prompt and effective case management with artemisinin-based combination therapy were the four key malaria control interventions [4, 5]. The protective efficacy of insecticide-treated nets (ITNs) results from both the physical barrier and the insecticidal action of the net. It provides protection to individual users and to the entire community through mass effect, when widely used [6]. ITNs can reduce the density, feeding frequency and survival of mosquitoes and wide-scale use can mediate protection of all community members, including those without a bed net [7, 8].

The use of ITNs in Ethiopia has started since 1997 and the scaling-up began in 2005 with the aim of obtaining a high coverage towards effective malaria control [9]. According to the central statistical agency (CSA) and Federal Ministry of Health of Ethiopia, 36 million bed nets, targeting about 52 million people at risk, were distributed between the period 2005 and 2010 [10, 11]. Thus, following the high coverage of LLINs and other preventive measures malaria incidence in the country has decreased by 50–75% in 2015 compared to 2000 [12]. However, the recent gains from malaria control could easily be unrolled back if the efficacy of LLINs is not regularly monitored and if insecticide resistance management strategies are not implemented.

PermaNet^®^ 3.0 is a deltamethrin-treated combination net with the addition of synergist piperonylbutoxide (PBO) on the roof section of the net [13]. Recently, the vector control advisory group of the World Health Organization (WHO) supported Vestergaard’s claim that relative to pyrethroid-only LLINs, the combination net, PermaNet^®^ 3.0 increased efficacy against malaria vector populations with cytochrome P450-based metabolic pyrethroid resistance, even if co-existing with *kdr* in a malaria vector population [14].

In Ethiopia, widespread insecticide resistance has been reported in the main malaria vector, *Anopheles arabiensis* and in few cases in *Anopheles pharoensis* [15]. This resistance is due to both target site and metabolic mechanisms [16, 17]. Although LLINs in general and PermaNet^®^ 3.0 in particular is currently being distributed for malaria vector control, their performance should be monitored in the field in different eco-epidemiological settings to assess their durability and long-term effectiveness for malaria prevention and control. Thus, the aim of this study was (1) to assess the susceptibility status of malaria vectors and nuisance Culicine mosquitoes against pyrethroid insecticides (deltamethrin and permethrin), (2) to evaluate the bio-efficacy of PermaNet^®^ 3.0 using the WHO cone bioassay, and (3) to evaluate the durability of PermaNet^®^ 3.0 after 3 years of use.

## Methods

### Study area and period

This study was conducted in two districts (Omo-Nada and Tiro-Afeta) in Jimma, zone, southwestern Ethiopia from March 2015 to August 2015. Anopheline mosquito larvae were collected from villages located in Tiro-Afeta and OmoNada districts (*weredas*) in south-western Ethiopia, from March to August 2015. Tiro-Afeta and Omo-Nada districts are located approximately 255 to 297 km southwest of the capital Addis Ababa [18]. The geographic coordinates of the districts lies between latitudes 7°42′50″N and 07°53′50″N and between longitudes 037°11′22″E and 037°20′36″E. Elevation of the districts range between 1672 and 1864 m above sea level. Both districts are known by sub-humid, warm to hot climate, receive between 1300 and 1800 mm of rain annually and a mean annual temperature of 19 °C [18]. The rainfall pattern of the area is like other parts of Ethiopia, with the long rainy season starting in June and extending up to September while the short rainy season begins in March and extends to April/May. The main socio-economic activities of the local communities are mixed farming involving the cultivation of staple crops (maize, teff and sorghum), and cattle and small stock-raising. Previous entomological studies conducted in the districts showed that *An. arabiensis* was the predominant species present in the area, and mosquito populations from both sites exhibited high resistance to DDT and pyrethroids (permethrin and deltamethrin). Moderate resistance to malathion (60.0–81.8% mortality) but susceptibility to propoxur (99.1–100% mortality) was also documented and very high (95–100%) allelic frequencies of kdr-L1014F mutation was reported in both populations [16].

### Study design

#### Mosquito rearing

Both Anopheline and Culicine mosquito larvae were collected by dipping from a range of breeding habitats (road paddies, brick pits, pools, marshes, streams, surface water harvest, ditches, dam reservoir shore, and pits dug for plastering traditional tukuls) located in Asendabo (Omo-Nada) and Kajelo (Tiro-Afeta) districts. The collected larvae were then brought to Jimma University Tropical and Infectious Diseases Research Center (TIDRC) insectary, Sekoru campus, under standard conditions following mosquito rearing guidelines [19]. Adults emerged from pupae were provided with 10% sucrose solution until the bioassay test.

#### Insecticide susceptibly test

Mosquito insecticide susceptibility level to pyrethroids (permethrin and deltamethrin) was tested using WHO tube test. Three to five days old, non-blood-fed female Anopheles were exposed to deltamethrin (0.05%) and permethrin (0.75) insecticide-impregnated papers. Involvement of possible metabolic resistance was assessed through pre-exposure of mosquitoes to synergist piperonyl butoxide (PBO) and then to the insecticide-impregnated papers, following the WHO standard assay [20]. The insecticide-impregnated, and control papers was obtained from the WHO Collaboration Centre, Vector Control Research Unit, School of Biological Sciences, University of Malaysia, Penang, Malaysia. Batches of 20–25 mosquitoes (in four replicates) were exposed to deltamethrin and permethrin impregnated papers in WHO tube for 1 h, and knockdown was recorded at 60 min. An equal number of mosquitoes (in two replicates) were exposed to the corresponding control papers impregnated with silicon oil (pyrethroid control). For synergist assays both insecticides were also run following a 1-h pre-exposure in which mosquitoes were introduced to four tubes containing the synergist piperonylbutoxide (PBO) impregnated paper for 1 h before undergoing the standard WHO susceptibility test.

### Bio-efficacy test of PermaNet^®^ 3.0

#### LLINs sample preparation and WHO cone assays

Three rectangular PermaNet^®^ 3.0 nets, were randomly selected from 75 used net samples collected from Omo-Nada and another three rectangular PermaNet^®^ 3.0 nets from Tiro-Afeta districts. Concurrently, six unused PermaNet^®^ 3.0 nets of the same batch were obtained from Asendabo Health Center. Untreated nets used as a negative control were purchased from the local market in Jimma, Ethiopia. The production date and batch number of all nets were recorded. Five sub-sample of nets (25 cm × 25 cm size) were cut from each net (four sub-samples from side panels and one sub-sample from roof panel) and prepared for standard LLINs cone test. Each sub-sample was rolled up in aluminum foil, labelled (by net type, net number and sample area) and kept individually in a refrigerator prior to the assay.

For each individual sub-sample, four cone tests were conducted following the standard WHO procedure [21]. Five non-blood-fed, 2–3 days old, female mosquitoes were introduced into each cone and exposed to each net sub-sample for 3 min before being transferred to paper cups and provided with 10% sugar solution. Knockdown (KD) was recorded at 60 min and mortality (MT) was recorded 24 h post-exposure. A total of 100 mosquitoes were tested for each net type (20 mosquitoes × 5 subsamples). For cone assay test, for every five sub-samples tested from one permanent 3.0 net, one sub-sample of untreated net was also tested concurrently as a negative control. Bioassays was carried out at a temperature of 27 ± 2 °C and relative humidity of 80 ± 10%.

#### Wash resistance

The wash resistance of LLIN was determined through standard bioassays carried out on nets washed at intervals using the standard WHO wash procedure, and dried and held at 30 °C. Bioassays were done after 0, 1, 5, 10, 15 and 20 washes. Each bioassay was done just before the next wash [21].

#### WHO washing procedure

Net sub-sample (25 cm × 25 cm) was individually introduced into a beaker containing 0.5 l deionized water, with 2 g/l soap (Savon de Marseille) at pH 10–11. Beaker was immediately kept in a water bath adjusted at 30 °C and shaken (155 movements per minute) for 10 min. The sub-samples were then removed and rinsed with deionized water twice for 10 min. Nets were then dried at room temperature and stored at 30 °C between washes in the dark box.

### Community based study of net utilization

#### Net sampling

A cross-sectional study was conducted to assess net physical integrity and utilization in 150 selected households from two districts (Omo-Nada and Tiro-Afeta), in March 2015. A total of 50,678 and 26,563 households were recorded in Omo-Nada and Tiro-Afeta districts, respectively [22]. From each district one village (Asendabo from Omo-Nada and Kajelo from Tiro-Afeta) was purposefully selected considering the malaria prevalence, the net brand of our interest and accessibility. Burka-Asendabo and Kajelo villages have a total population of 7080 and 4270 with 1500 and 850 households, respectively. Then from each village 75 households (total of 150) were randomly selected for this study. The name and location of the randomly selected households were identified from the list of households in health post in consultation with health extension worker. The two districts were the sites in Jimma zone where PermaNet^®^ 3.0 (with batch number 3.0 101213) had been distributed 3 years before study. The distribution of LLINs to each household was done by National Malaria Control Programme (NMCP) with the support of PMI-USAID according to the programme’s net distribution guidelines (one net for two persons) [10]. One net was collected from each of selected household by replacing with the new ones. Net survivorship evaluation was not possible, as we could not exactly know the number of nets distributed in each household, hence in this study only the net physical integrity, bio-efficacy and socio-demographic factors associated with net utilization were evaluated.

#### Socio-demographic data collection

Socio-demographic data such as educational status, housing condition, source of energy, source of pure water, presence of latrine, family size, sleeping place, net condition, net usage and handling were collected from each selected household using a semi-structured questionnaire. In addition, information pertaining to net hanging practice, sleeping place, flooring of the house, wall of the house, ceiling of the house, net storage condition and the number of holes (if present) per each net were also recorded.

### Evaluation of physical integrity of net

Nets were individually hanged over a rectangular 180 cm × 180 cm × 180 cm wooden frame. Hole, seam failure and repair were recorded. The size and location of each hole was recorded for each net and the hole size was measured as the long axis of the ellipse to the nearest cm. Only holes greater than 0.5 cm were counted and recorded. Hole location was recorded separately for (Roof, side and seam) and hole size was measured with ruler by categorizing as size 1 (0.5–2 cm), size 2 (2–10 cm), size 3 (10–25 cm), size 4 (greater than 25 cm) following standard protocol [21]. Proportionate hole index (pHI) was employed to assess the physical quality of each net by weighting the size of each hole and summing them for each net according to the WHO protocol [21]. Nets were then classified as ‘good’, ‘acceptable’ and ‘too torn’ depending on the pHI value. Thus, those with pHI between 0 and 64 were considered as being in ‘good’ condition and there should be no reduction of efficacy compared to an undamaged net. The second net category were those with pHI between 65 and 642. They were considered as ‘acceptable’ despite their effectiveness was moderate compared to undamaged nets but provide significant protection compared to absence of net. The third category is nets classified as ‘too torn’ with pHI equal or > 643 where the protective efficacy for the user is in serious doubt and the net should be replaced as soon as possible.

### Data analysis

Mosquito knockdown and mortality data were analyzed and presented using frequency and percentage table. Proportionate hole index (pHI) was calculated using the formula pHI = (1 × no. of size-1 holes) + (23 × no. of size-2 holes) + (196 × no. of size-3 holes) + (578 × no. size-4 holes) [21]. Binary logistic regression model was fit to determine factors associated with observed physical damage of nets. Crude odds ratio with 95% confidence interval were reported and P < 0.05 were considered significant during the analysis.

### Ethics approval and consent to participate

This study was first reviewed and approved by institutional review board (IRB) of College of Naturals Sciences, Jimma University. Then the University wrote support letter to Zonal and District Health Bureau to get consent and for net replacement. Finally, signed consent was obtained from each head of the household participated in the study.

## Results

### Socio-demographic characteristics

Socio-demographic status of community members participated in the study area is presented in Table 1. Lack of proper education remained deep-rooted problem of the community as majority of the household heads 122 (81.3%) were either illiterate 58 (38.7%) or simply attended religious school 64 (42.6%). The rest 25 (16.6%) attended primary education and very few 3 (2%) respondents attended secondary school. Access to electricity was very low as majority of the households 113 (75.3%) had no electricity and only 37 (24.7%) had electricity in their residences. Access to clean drinking water remained low as 83 (55%) of the study participants reported use of spring water and another 22 (14.7%) reported either use of surface water or unprotected public well. Most community members 142 (94.7%) used private pit latrine and over half of the respondents 88 (58.7%) reported the family size of five and more.

**Table 1:** Socio-demographic characteristics of respondents in Omo-Nada, Tiro-Afeta districts Southwestern Ethiopia (August 2015)

Variables	Respondents	n (%)
Education	None	58 (38.7)
	Religious	63 (42)
	Primary	26 (17.3)
	Secondary	3 (2)
Access to electricity	Yes	37 (24.7)
	No	113 (75.3)
Source of drinking water	Protected public well	45 (30)
	Unprotected public well	15 (10)
	Surface water	7 (4.7)
	Spring water	83 (55.3)
Latrine	Own pit latrine	142 (94.7)
	Bush or field or other	8 (5.3)
Family size	1–2	30 (20)
	3–4	32 (21.3)
	Above 5	88 (58.7)

### WHO insecticide susceptibility test

Results of WHO insecticide susceptibility test against wild populations of *Anopheles gambiae* sensu lato (presumably *Anopheles arabiensis*; [16]) and *Culex quinquefasciatus* are presented in Table 2. The exposure of adult *An. gambiae* s.l. to permethrin resulted in 15% knockdown and 39% mortality and exposure of *Culex quinquefasciatus* to permethrin resulted in 10% knockdown and 28% mortality. Similarly, deltamethrin impregnated papers caused 32% knockdown and 52% mortality against *An. gambiae* s.l. and 25% knockdown and 43% mortality against *Culex quinquefasciatus*. However, following pre-exposure to synergist PBO the susceptibility of mosquito population increased to both permethrin (from 39% without to 92% with PBO against *An. gambiae* s.l. and from 28% without to 94% with PBO against *Culex quinquefasciatus*) and deltamethrin (from 52% without to 99% with PBO against *An. gambiae* s.l. and from 43% without to 98% with PBO against *Culex quinquefasciatus*).

**Table 2:** Susceptibility level of *Anopheles gambiae* and *Culex quinquefasciatus* to pyrethroids and pyrethroid-synergist combination in Jimma Zone Ethiopia (2015)

Mosquito spp.	Insecticide	# tested	Knockdown (%)	Mortality (%)
*An. gambiae*	Permethrin	100	15	39
	Deltamethrin	100	32	52
	Permethrin + PBO	100	94	92
	Deltamethrin + PBO	100	96	99
	Control	100	1	2
*Cx. quinquefasciatus*	Permethrin	100	10	28
	Deltamethrin	100	25	43
	Permethrin + PBO	100	90	94
	Deltamethrin + PBO	100	95	98
	Control	100	2	5

### Bio-efficacy of unused PermaNet^®^ 3.0 nets against wild populations of *Anopheles gambiae* and *Culex quinquefasciatus*

**Fig. 1:**
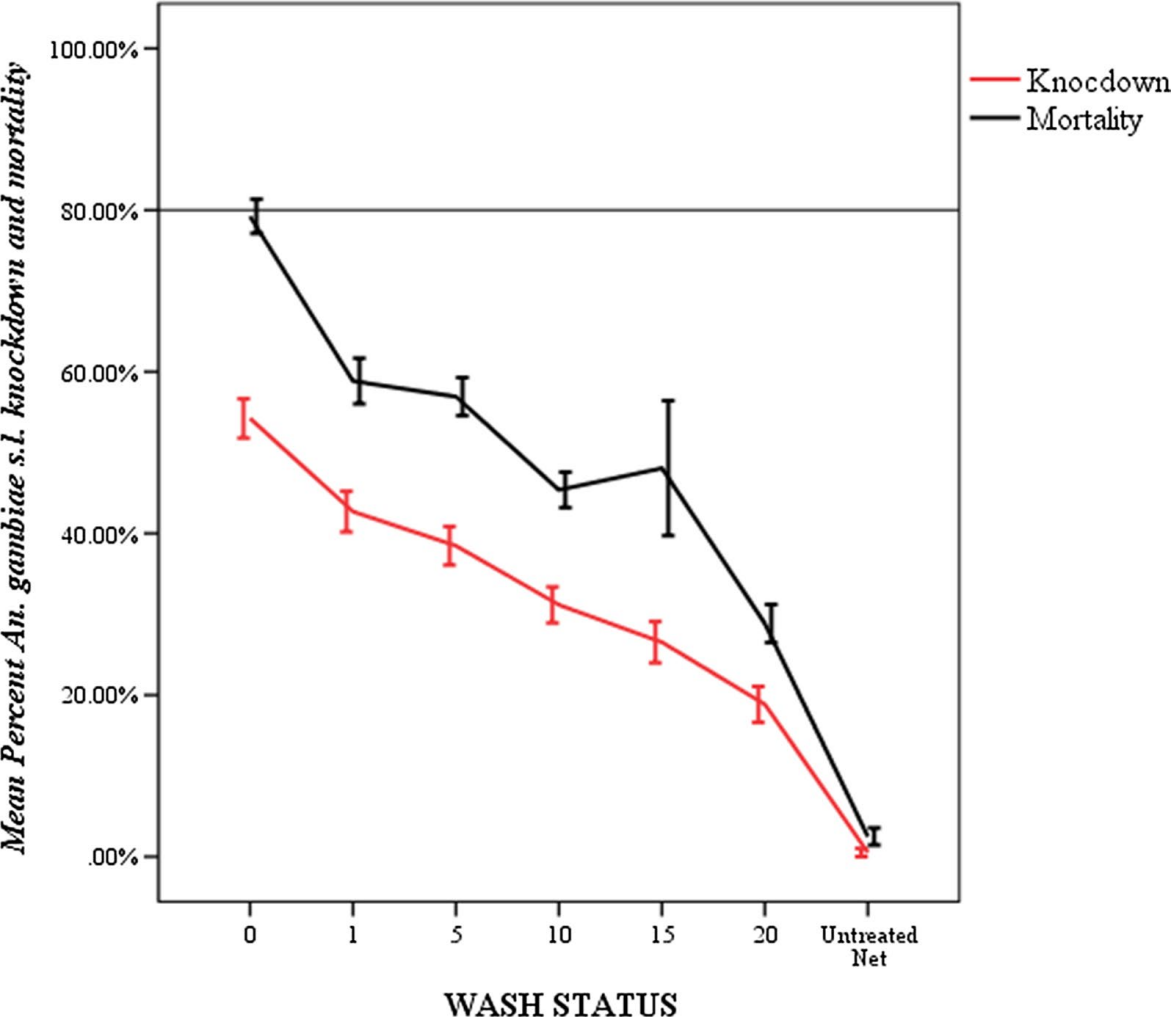
Mean percentage knockdown and mortality rates of *An. gambiae* s.l. exposed to unused PermaNet^®^ 3.0 nets (August 2015)

The mean percent knockdown and mortality of unused PermaNet^®^ 3.0 nets against wild population of *An. gambiae* is presented in Fig. 1. Mean percent knockdown and mortality recorded when mosquitoes were exposed to unwashed nets were 54% and 80%, respectively. Both mean percent knockdown and mortality declined as washing frequency increased from 0 to 20. Accordingly, the least knockdown and mortality rates, 19% and 20%, respectively, were recorded when mosquitoes exposed in nets washed 20 times. There was significant difference in mean mosquito knockdown and mortality rates among net washes (P < 0.001).

**Fig. 2:**
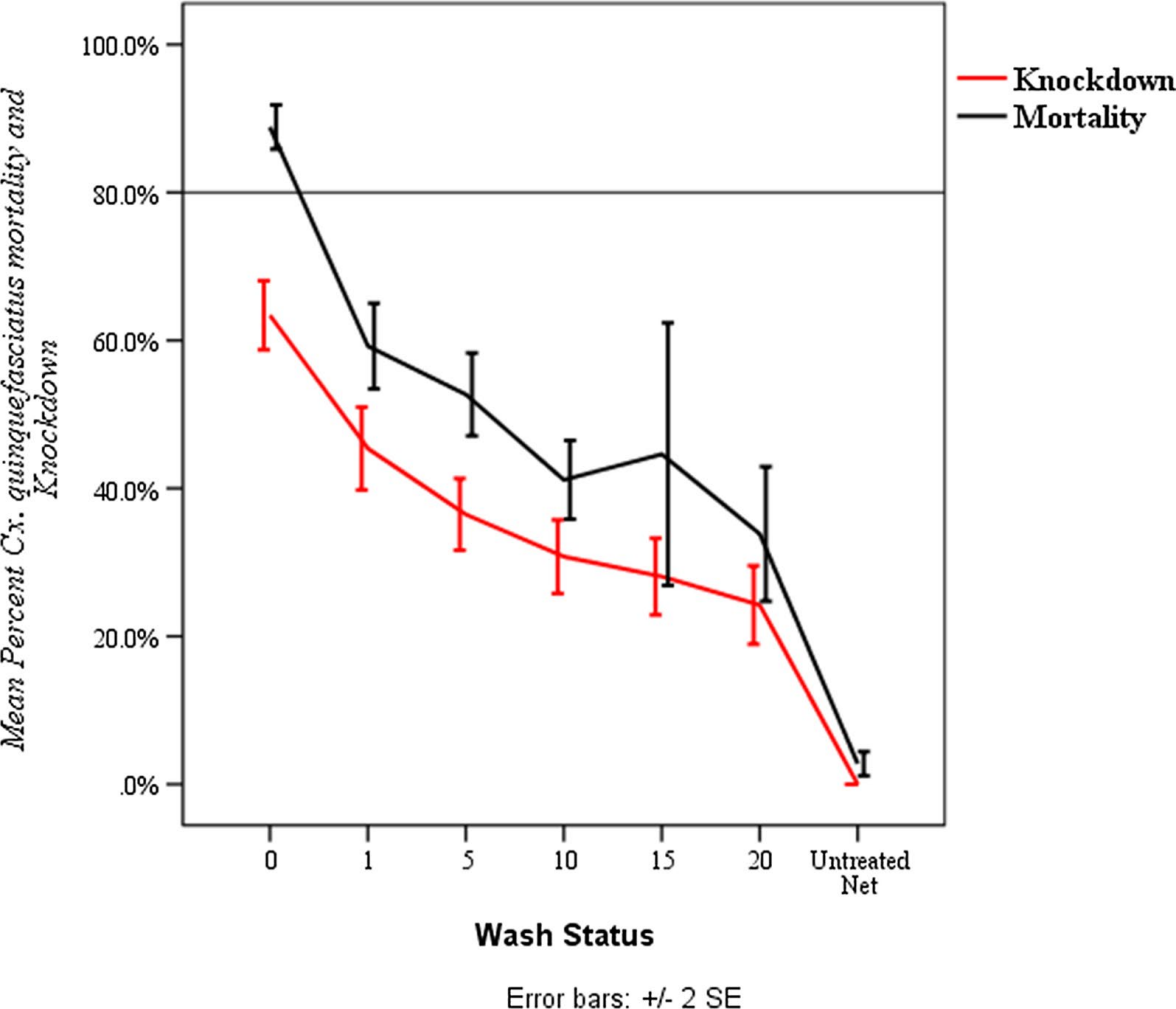
Mean percentage of knockdown and mortality rates of *Cx. quinquefasciatus* exposed to unused PermaNet^®^ 3.0 (August 2015)

The mean percent knockdown and mortality of unused PermaNet^®^ 3.0 nets against wild population of *Culex quinquefasciatus* is presented in Fig. 2. The highest mean knockdown and mortality rates (63% and 89%, respectively) were recorded when *Culex quinquefasciatus* were exposed to unwashed PermaNet^®^ 3.0 nets. Both mean knockdown and mortality rates were declined as washing frequency increased from 0 to 20. Accordingly, the least mean percent knockdown and mortality (24% and 34%, respectively) was recorded when mosquitoes were exposed to net washed 20 times. There was a significant decline in mean percent knockdown and mortality among net washes (P < 0.001).

**Fig. 3:**
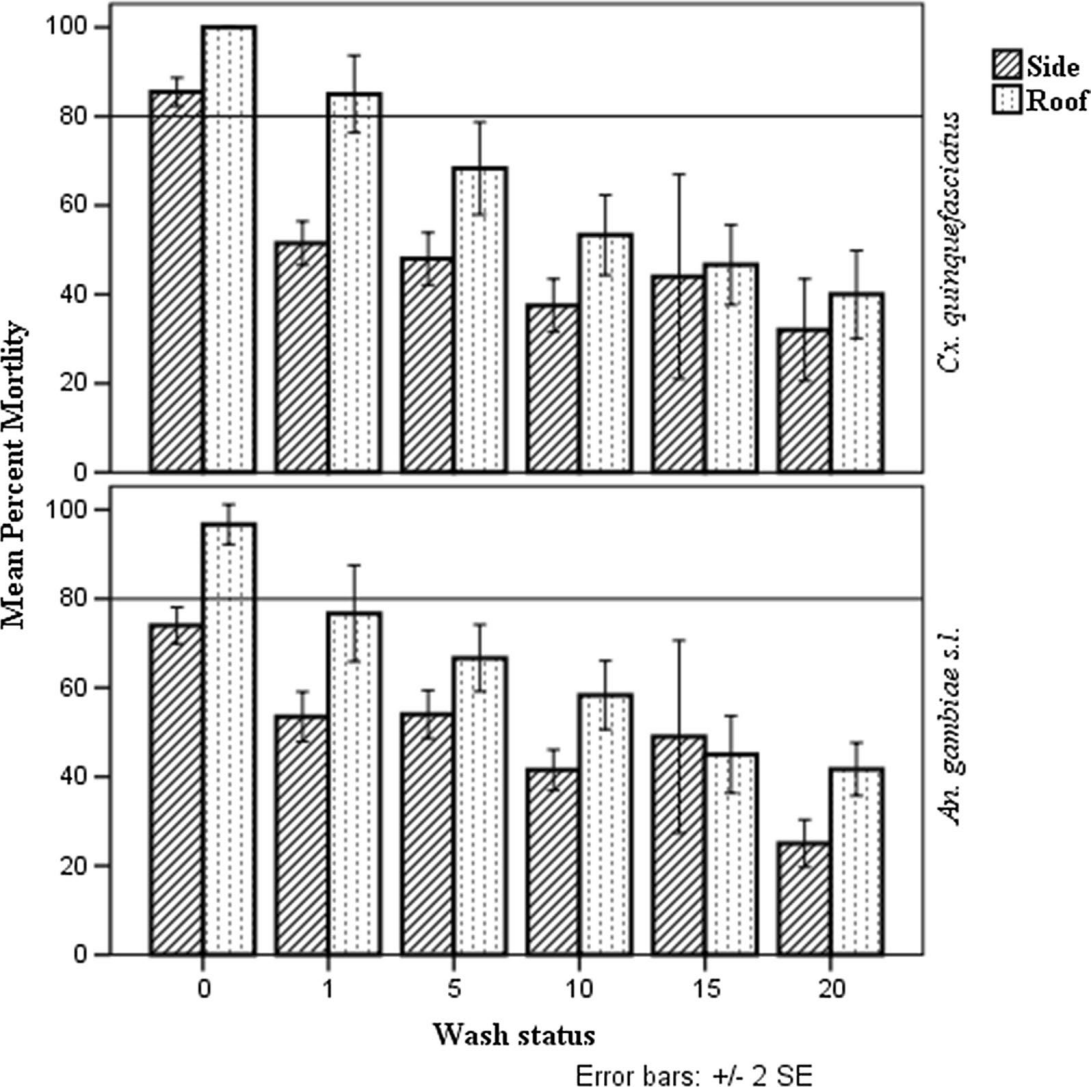
Mean percentage knockdown and mortality rates of *An. gambiae* s.l. and *Cx. quinquefasciatus* exposed to roof and side panels of unused PermaNet^®^ 3.0 net (August 2015)

There was variation in mean percent mortality of *An. gambiae* s.l. and *Culex quinquefasciatus* exposed to roof and side panels of PermaNet^®^ 3.0 (Fig. 3). Percent mortality of *An. gambiae* and *Culex quinquefasciatus* exposed to the side panels section before washing was 74% and 85%, respectively. Its bio-efficacy started to significantly decline (P < 0.001) immediately after the first wash with percent mortality of *An. gambiae* and *Culex quinquefasciatus* going down to 25% and 32%, respectively after 20 washes. The roof section however, induced higher percent mortality than the side section. It resulted in 97% and 100% mortality against *An. gambiae* and *Culex quinquefasciatus,* respectively prior to net washing. Similarly, the performance of the side section started to decline significantly (P < 0.001) following 20 washes with mortality of 41% and 40% for *An. gambiae* and *Culex quinquefasciatus*, respectively.

### Bio-efficacy of used PermaNet^®^ 3.0 nets against susceptible strain of *Anopheles arabiensis* after 3 years of use

**Fig. 4:**
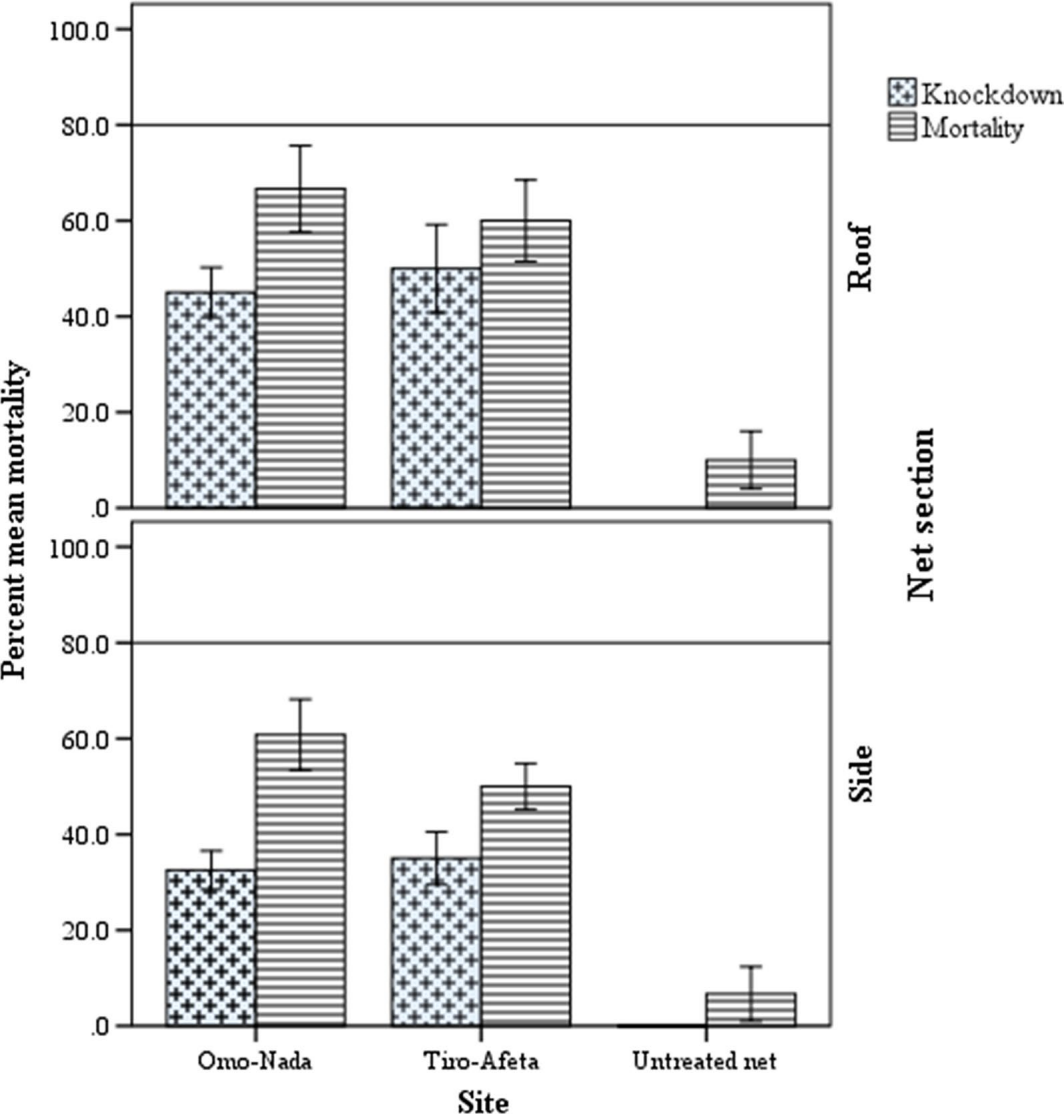
Mean percentage knockdown and mortality rates of wild populations of *An. gambiae* s.l. exposed to different sections of used PermaNet^®^ 3.0 nets, Ethiopia

The mean percent knockdown and mortality of susceptible strains of *An. arabiensis* exposed to the roof and side panels of used PermaNet^®^ 3.0 nets are presented in Fig. 4. Mean percent knockdown and mortality of *An. arabiensis* exposed to side sections of used PermaNet^®^ 3.0 sub**-**samples resulted in 55.8% and 82.5% for Omo-Nada and 69.2% and 80.8% for Tiro-Afeta, respectively. Moreover, mean percent knockdown and mortality of *An. arabiensis* exposed to roof sections of used PermaNet^®^ 3.0 sub-samples were 68.3% and 90% for Omo-Nada and 73.3% and 95.0% for Tiro-Afeta, respectively. There was significantly higher mean percent knockdown (P < 0.005) of *An. arabiensis* exposed to nets collected from Tiro-Afeta than mean percent knockdown to nets collected from Omo-Nada district. However, there was no variation in mean percent mosquito mortality from nets collected from the two districts (P = 0.983).

### Bio-efficacy of used PermaNet^®^ 3.0 nets against wild populations of *Anopheles arabiensis* after 3 years of use

**Fig. 5:**
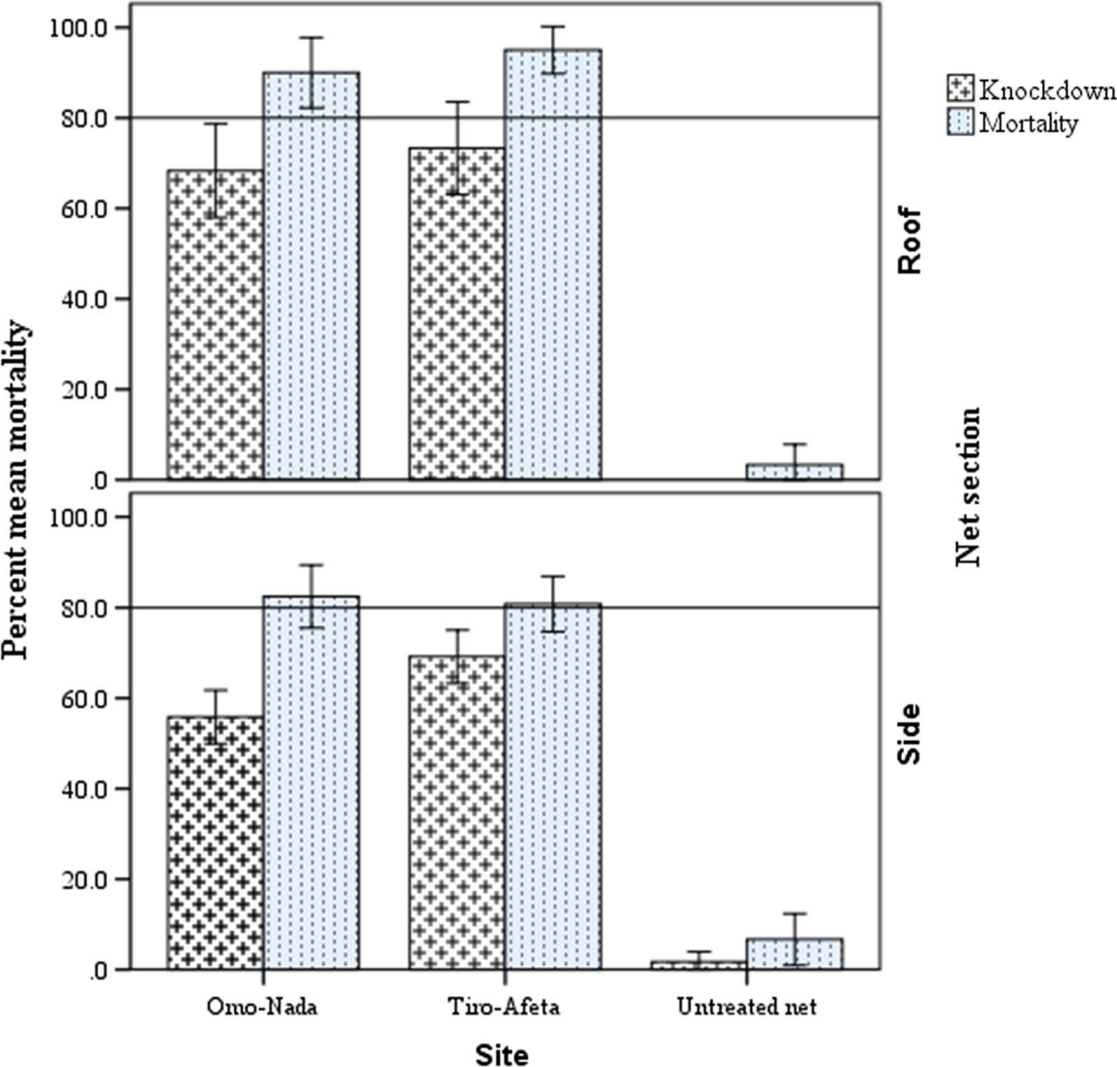
Mean percentage knockdown and mortality rates of laboratory strain of *An. arabiensis* exposed to side and roof panels of used PermaNet^®^ 3.0 nets Ethiopia

The mean percent knockdown and mortality of wild population of *An. gambiae* s.l. exposed to used PermaNet^®^ 3.0 nets collected from Omo-Nada and Tiro-Afeta are presented in Fig. 5. Exposure of *An. gambiae* to side sections of PermaNet^®^ 3.0 sub**-**samples collected from Omo-Nada and Tiro-Afeta districts after 3 years of use resulted in mean percent mosquito knockdown and mortality of 32.5% and 60.8% for Omo-Nada and 35.0% and 50.0% for Tiro-Afeta, respectively. Similarly, exposure of *An. gambiae* to roof sections of PermaNet^®^ 3.0 sub-samples collected from Omon-Nada and Tiro-Afeta districts resulted in mean percent mosquito knockdown and mortality of 45.0% and 66.6% for Omo-Nada and 50.0% and 60.0% Tiro-Afeta, respectively. There was no significant variation in bio-efficacy between net sub-samples collected from the two districts (P = 0.309). However, the available bio-active chemical on the surface of LLINs after 3 years of usage was clearly displayed as there was significantly higher mean percent mosquito knockdown and mortality (P < 0.001) as compared to untreated nets.

### Net possession, handling and usage

A total of 150 heads of households were interviewed from Omo-Nada and Tiro-Afeta districts. In this survey, all households (100%) possessed at least one LLIN at the time of the survey and 147 (98%) of the LLINs collected from each household were used for sleeping purpose prior to the collection day. Moreover, 126 (84%) of the respondents reported that they used the nets most often. In households where nets were scarce priority was given to children and all children (100%) in the study area were protected. The majority of the respondents 120 (80%) had more than one type of sleeping place at home (Wooden bed frame, wooden bed stick, and mattress placed on carpet on bare floor). One hundred five (69%) of the respondents reported that they practiced net washing in the past and 84 (56%) of them washed in the last 6 months prior to net collection. Sixty-one percent of the respondents washed their net with local bare soap and dried their net outdoor in the shade.

### Physical integrity of LLINs and factors contributing to net damage

Of 150 LLINs collected from the study districts, most of the nets were found hanged over the sleeping places in different forms; hanged folded 57 (38%), hanged tied 56 (37.3) or hanged lose 34 (22.7%). Very few nets 3 (2%) were found stored. Most of the nets 144 (96%) were found indoor and only 6 (4%) were found outdoor. Housing conditions were overwhelmingly of typical traditional type with 147 (98%) of the houses with mud floor and wall made from mud with wooden frame. Ninety-six (64%) of the houses were thatched roof houses and 54 (36%) of were corrugated iron roof houses. Most of the respondents 148 (98.6%) reported that open flame from firewood was the main source of power for cooking and used oil lump during the night as source of light.

The impact of household factors such as housing style, cooking and washing habits, misuse by children and damage due to domestic pests, such as rodents, on physical integrity of bed nets is presented in (Table 3). Accordingly, frequently washing nets, residing in houses with thatched roof, using fire for cooking and the presence of domestic pests such as rodents are the most prevalent reported cause of bed net physical damage.

**Table 3:** Association of net physical integrity and household factors

Variables	Response	Physical condition of net		COR (95% CI)	P-value
		Without hole n (%)	With hole n (%)		
Net ever been washed	Yes	7 (4.7)	67 (44.7)	5.3 (2.12–13.10)	< 0.001
	No	27 (18)	49 (32.7)		
House type	Thatched roof	15 (10)	81 (54)	2.9 (1.34–6.42)	0.007
	Iron sheet	19 (12.7)	35 (23.3)		
Fire and oil lump used for cooking and lighting	Yes	6 (4)	85 (56.7)	12.8 (4.84–33.85)	< 0.001
	No	28 (18.7)	31 (20.7)		
Misuse by children	Yes	24 (16)	93 (62)	1.69 (0.71–4.01)	0.239
	No	10 (6.7)	23 (15.3)		
Net damage due to rodents	Yes	24 (16)	103 (68.7)	3.30 (1.29–8.42)	0.012
	No	10 (6.7)	13 (8.7)		

### Determination of net hole and size

**Fig. 6:**
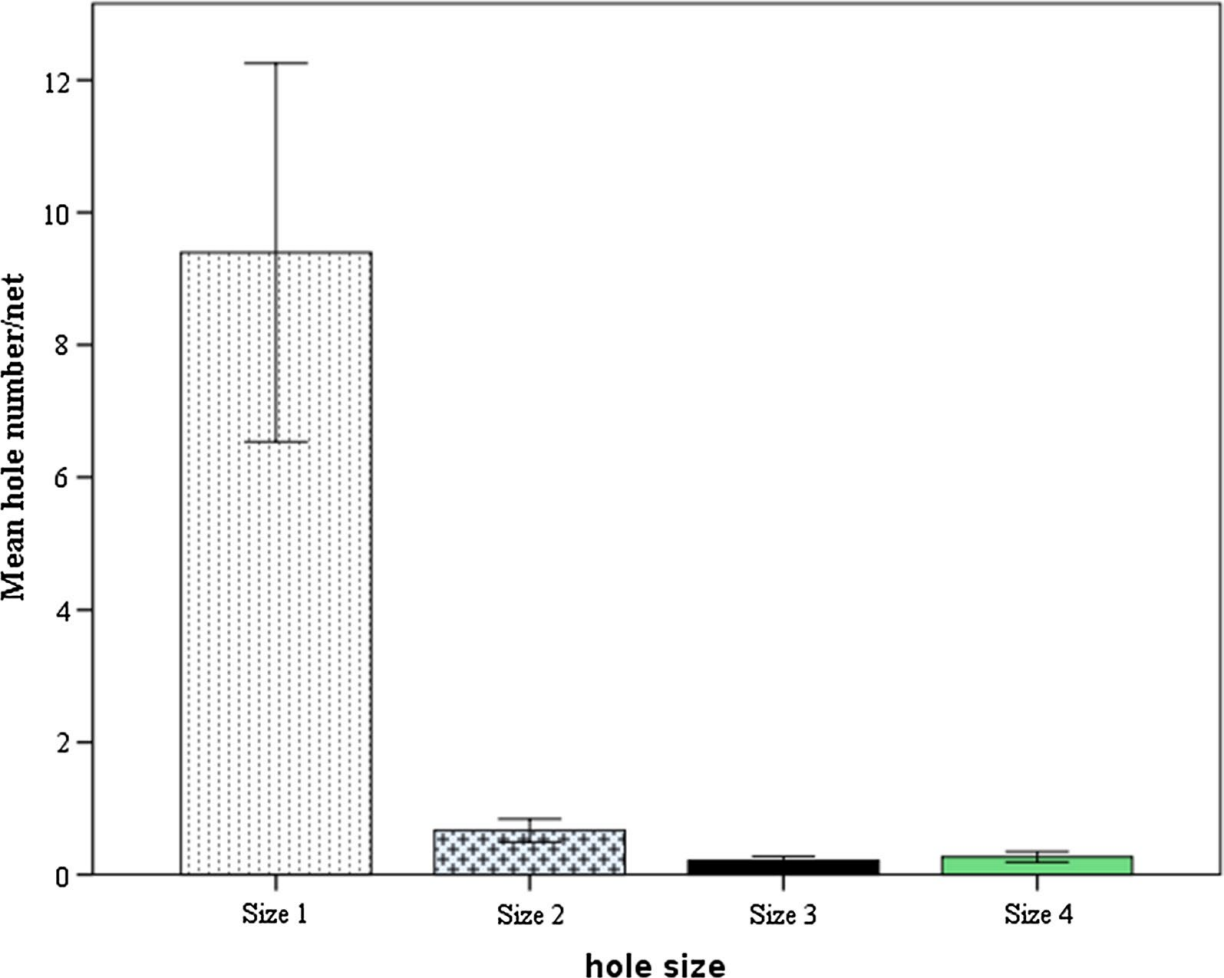
Mean number of holes per net after 3 years of use in Ethiopia

Of 150 LLINs collected from the community, 116 (77%) were with different number and size of holes whereas the rest 34 (23%) were intact. In total, 4743 holes were recorded from nets collected from communities after 3 years of use. The mean number of holes was 31.7 per net. Of the total 4743 holes, 2869 (60.3%) holes were recorded from side panels, 1554 (32.6%) were recorded from roof section and the rest 337 (7.1%) were recorded from seams. Of the total 4743 holes, 4228 (89%) were size 1, 300 (6.5%) were size 2, 95 (2%) were size 3 and 120 (2.5%) were size 4 (Fig. 6). Sixty-two (41%) of the nets were with horizontal tear at the bottom, 93 (62%) were with tear at hanging points, 81 (54%) of the nets were with burn holes. Thirty-four of the nets were with open seam with whole section missing. The proportionate hole index (pHI) ranged from 0 to 6064. Accordingly, 80 nets were in ‘good’ condition (pHI ranged 0 to 64), 29 nets were ‘acceptable’ (pHI ranged, 65 to 642) and 41 nets were ‘too torn’ (pHI > 643).

## Discussion

According to the WHO framework for malaria elimination, the success in achieving the objective of the SDG 3.3 global targets for malaria depends on public awareness about the value of human health, the use of nets and the provision of effective access to nets [1]. In line with this, the NMCP of Ethiopia currently relies on strategies targeting mosquito vector control, which involves the use of LLINs, IRS and larval source management [12]. In this study, the emergence of pyrethroid resistance in Culicine and Anopheline mosquitoes against permethrin and deltamethrin (the two commonly used pyrethroids in net impregnation) was assessed in two districts of Jimma zone, Southwest Ethiopia. Furthermore, net bio-efficacy against local mosquito populations of *Anopheles* and *Culex* was evaluated. In addition, net possession, handling and usage was assessed through community based cross-sectional survey in Omo-Nada and Tiro-Afeta districts, Southwestern Ethiopia.

Results of the susceptibility test using wild *An. gambiae* s.l. (presumably *An. arabiensis*; [16]) and *Culex quinquefasciatus* against WHO insecticide-impregnated papers showed that both permethrin and deltamethrin resulted in reduced knockdown and mortality. However, pre-exposure of mosquitoes to synergist PBO resulted in higher mortality for both insecticides. Similar findings were reported by Yewhalaw et al. [18] in that pre-exposure of pyrethroid resistant *An. arabiensis* mosquitoes collected from Jimma area, southwestern Ethiopia for 1 h to PBO, increased the susceptibility of mosquitoes in all four sites to deltamethrin (mortality range 91.8 to 100%) and permethrin (mortality range 73.9 to 100%). Moreover, the vector population in the same area developed multiple resistance for three (organochlorines, pyrethroid and organophosphates) insecticides out of the four classes of insecticides recommended for public health application [16]. This report on pyrethroid resistance populations of *Culex quinquefasciatus* from Jimma area, is the first report in Ethiopia.

In this study, unwashed PermaNet^®^ 3.0 induced more than 80% mortality, the WHO threshold for standard product efficacy, against wild population of *An. gambiae* and *Culex quinquefasciatus* However, reduced knockdown effect was recorded against both *Culex* and *Anopheles* population. Moreover, the efficacy and wash resistance rapidly downgraded as the number of washes increased from 0 to 20. Reduced knockdown effect of unwashed PermaNet^®^ 3.0 against pyrethroid resistant populations of both *An. gambiae* and *Culex quinquefasciatus* is the first report from Ethiopia. Other bio-efficacy tests conducted in Nigeria [23], Mozambique [24], Tanzania [25], Côte d’Ivoire [26], Central and western Africa [27] showed susceptibility of wild populations to the new brand PermaNet^®^ 3.0 net with knockdown effect ranging from 95 to 100%. The observed decline in bio-efficacy of PermaNet^®^ 3.0 in the current study after successive washes is also in consistent with findings from Côte d’Ivoire [26] and Tanzania [28], which also reported that the bio-efficacy of PermaNet^®^ 3.0 declined from 100% mortality when tested at 0 wash to 4 to 15% after 20 washes.

The discriminate killing effect of the roof section and side panels of PermaNet^®^ 3.0 was evidenced when unwashed PBO-deltamethrin top netting induced significantly higher mean mortality in both *An. gambiae* and *Culex quinquefasciatus* and compared to the side section. The enhanced killing effect of PBO-deltamethrin coated top netting of PermaNet^®^ 3.0 is well documented from studies conducted in Ethiopia [18] and Tanzania [28] which showed significantly different mosquito mortalities between roof section and side panels against *An. gambiae* and *Culex* spp.

Used PermaNet^®^ 3.0 collected from both districts after 3 years of deployment resulted in moderate to low bio-efficacy, below WHO threshold against field collected *An. gambiae,* but exposure of susceptible strains of *An. arabiensis* to same net samples resulted in mean knockdown and mortality rate above the WHO cut-off point. Comparison of mean knockdown and mortality rates of side and roof sections of the same samples showed enhanced killing effect of PBO coated roof section, which produced greater mortality compared to side panels of the net. The observed low knockdown and mortality against both wild *An. gambiae* and laboratory-reared susceptible strains of *An. arabiensis* could be due to removal of surface concentration of PBO by human activities, such as washing, rubbing, smoking and overutilization. Similar finding was reported from Kenya by Ochomo et al. [29], which stated that nets collected from field retained strong activity against a susceptible laboratory strain, but not against f1 offspring of field-collected *An. gambiae*.

In this study, at least one LLIN was traced in each of the 150 houses included in the survey. While it was not possible to corroborate the universal coverage of ITN in this survey since we could not determine the exact number of nets distributed at the beginning of the campaign, it was apparently clear that the districts managed to reach 100% target of one ITN per household. Moreover, most of the LLINs used for sleeping purpose prior to the collection day. This was a good trend of LLINs coverage in comparison to national coverage of 64% households with at least one LLINs [30]. The current coverage was also greater than findings from Amhara region, Ethiopia by Aderaw and Gedefaw [31] who reported that 84.67% of the households possessed functional bed nets, and 71.4% of them have been slept under bed net a day before the interview took place. Likewise, high LLIN ownership and usage rate was reported from Amhara and Oromia regional states, Ethiopia with 91% of the respondents own at least one ITN prior to the survey date [32]. The high ITN coverage is not uniform throughout the country. For instance, low ITN ownership and usage rate (62% and 65%) was reported from Eastern Ethiopia by Biadgilign et al. [33], Gobena et al. [34], and from western Ethiopia by Tadele et al. [35] whom documented 69.3% coverage and 64.9% utilization rate. This could be due to priorities given to the severely affected areas by the NMCP.

In this study, nets were washed on average once every 6 month as more than half of the respondents cited it. Most respondents washed their net with local bar soap and dried outdoor under shade. Frequently washing nets, residing indoor with thatched roof, smoking using fire and oil for cooking and the presence of domestic pests such as rodents are the factors attributed to reported net damage and worn-out problem in the study area. Other studies also indicated the effect of smoke from wood fire used in cooking in the same home where bed nets were hanged in contributing to the observed net damage and reduced efficacy [36]. Children playing with sharp objects near nets, attack from rodents, and damage from sleeping mat are primary source of net damage according a study from Nigeria [37].

In this study, most nets collected from the community after 3 years of use were with variable number and size of holes. Most of the holes were small and documented from seam, side, roof section and hanging points of the net. Most of the holes were from side section of the net. Damage recorded included horizontal tear at the bottom, tear at hanging points, burn holes, holes from rodents and the nets with open seam with the whole section missing. Based on the proportionate hole index (pHI) assessment of nets had varying degree of damage. Thus, most of nets were good or acceptable, however, 41 (27%) nets were too torn and need immediate replacement. The abundance of small holes in the side section of the net in other studies from Cameroon [38] and Mozambique [39] was documented.

## Limitations

Chemical analysis for both used and unused permanent 3.0 samples was not conducted due to logistic limitations which could have better explained the impact of household factors such as traditional washing, rubbing, smoking on bio-efficacy of bed nets. Net survivorship (attrition rate) evaluation was not possible, as the number of nets distributed were not exactly known. In addition to determining attrition rate prospective studies are critical in order to determine the onset of net deterioration during the intervention period.

## Conclusions

The susceptibility test using wild populations of *Culex quinquefasciatus* and *An. gambiae* against WHO insecticide-impregnated papers showed that both permethrin and deltamethrin resulted in reduced mortality and knockdown, however, pre-exposure of mosquitoes to synergist PBO resulted in higher mortality and knockdown for both permethrin and for deltamethrin confirming the involvement of metabolic enzymes in the observed resistance. The efficacy and wash resistance of PermaNet^®^ 3.0 rapidly decreased as the number of washes increased from 0 to 20 times. The exposure of mosquitoes to unwashed PBO-deltamethrin coated top netting induced the highest mortality in both *Culex* and *Anopheles* mosquitoes. The evaluation of PermaNet^®^ 3.0 LLIN showed moderate efficacy against pyrethroid resistant population of *An. gambiae* and *Culex quinquefasciatus* from Omo-Nada and Tiro-Afeta districts south western Ethiopia. Thus, timely implementation of insecticide resistance management strategy and integrated vector management remains critical action in order to slowdown the emergence of the observed insecticide resistance. The bio-efficacy of PermaNet^®^ 3.0 bed net samples collected from Omo-Nada and Tiro-Afeta districts after 3 years of service has resulted in reduced knockdown and mortality. All households in the study village possess at least one LLIN with majority of the LLINs were being used for sleeping purpose prior to the collection day. However, household problems such as hanging points, housing conditions, open flame fire used for cooking and rodent attack were identified as factors contributing to the observed bed net physical quality loss in the study area.

## Abbreviations

LLINs: long-lasting insecticidal nets
ITN: insecticide treated net
IRS: indoor residual spray
NMCP: National Malaria Control Programme
WHO: World Health Organization
PBO: piperonylbutoxide
pHI: proportionate hole index
